# Ulcerative colitis in a Nigerian girl: A case report

**DOI:** 10.1186/1756-0500-5-564

**Published:** 2012-10-10

**Authors:** Idowu O Senbanjo, Kazeem A Oshikoya, Charles A Onyekwere, Fatimah B Abdulkareem, Olisamedua F Njokanma

**Affiliations:** 1 Department of Paediatrics and Child Health, Lagos State University College of Medicine, PMB 21266, Ikeja, Lagos State, Nigeria; 2 Pharmacology Department, Lagos State University College of Medicine, PMB 21266, Ikeja, Lagos State, Nigeria; 3 Academic Division of Child Health, Medical School (University of Nottingham), Derbyshire Children’s Hospital, Uttoxeter Road, Derby, DE22 3DT, UK; 4 Department of Medicine, Lagos State University College of Medicine, PMB 21266, Ikeja, Lagos State, Nigeria; 5 Gastrointesinal/Hepato-pathology Unit, Morbid Anatomy Department, College of Medicine, University of Lagos, P.M.B. 12003, Idi-Araba, Lagos, Nigeria; 6 Paediatrics Gastroenterology, Hepatology and Nutrition Unit, Department of Paediatrics and Child Health, Lagos State University Teaching Hospital, Ikeja, Lagos, Nigeria

**Keywords:** Ulcerative colitis, Child, Nigerian

## Abstract

**Background:**

Ulcerative colitis (UC) is uncommon in the tropics and sub-tropics. We report a case of UC in a 7 year old girl whose parents were both Nigerians. This report is to alert healthcare professionals in sub-Saharan Africa that UC is not a rare health problem, especially in children.

**Case presentation:**

The patient presented with frequent passage of blood stained stool, abdominal pain and significant weight loss. The diagnosis was entertained after she was investigated for common causes of chronic diarrhea in our setting and the findings were negative. The patient symptoms abated after she was commenced on steroid therapy.

**Conclusion:**

Under-diagnosis and misdiagnosis may account for a dearth of information on UC in African children.

## Background

Ulcerative colitis (UC) is a chronic inflammatory disease of unknown aetiology, localized to the colon and spares the upper gastrointestinal tract [1]. The inflammation is characteristically remitting and relapsing [2].

The prevalence of UC varies across geographical zones and from one country to another [3]. In North America, the prevalence varies from 37.5 to 238 per 100000 people [4]. The disease is less common in children than adults [2]. In patients with UC, 20% are younger than 20 years of age, 4% are children aged less than 5 years and 1% are infants [3,5]. Although, UC can occur at any age, the incidence peak in age group 15–25 years and in 55–65 years [6]. The first paediatric case was reported in 1923 by Helmholz [7], thereafter, several other cases have been reported in children [8-10]. UC usually exists in isolation and together with Crohn’s disease (CD) and indeterminate colitis (IC) constitutes a genetically, immunologically and histopathologically heterogeneous group of inflammatory bowel disorders called inflammatory bowel disease (IBD) [11]. Very rarely are these diseases diagnosed in the same patient [12]. However, a rare case of UC co-exiting with CD had been reported in an adult [13].

Many familial cases have been reported in the United States, yet no simple Mendelian genetic mechanism has been able to explain its transmission in those with associated family history of the disease [2]. The prevalence of UC is highest in Europe and America among Caucasians and Ashkenazic Jews and lowest in black Americans and in African countries and Japan [1]. Although, cases of UC in African-American children living in the United States of America have been reported [14], none has been reported in Africa. The rarity of the disease in Africa may limit the experience of clinicians in its diagnosis and management, especially in children. This case is therefore reported to create an awareness of UC among paediatric age group and to discuss the challenges facing the diagnosis and management of the disease in a resource poor country.

## Case presentation

A 7 year old girl presented to the paediatric gastroenterology clinic at the Lagos State University Teaching Hospital (LASUTH), Ikeja with a history of prolonged diarrhoea of 10 weeks that progressed to frank haematochezia 2 weeks later. She also presented with abdominal pain weight loss of over 8 weeks duration. Stool was initially watery, not offensive or mucoid. Bowel motions were about 10 times per day. There was no vomiting, fever, jaundice, mouth ulcer or joint pains. The abdominal pain was crampy, diffusely localized to the umbilical and supra-pubic regions. It was neither aggravated nor relieved by any known factors. Pain did not radiate elsewhere nor, disturb the patient from sleep, associate with tenesmus or abdominal distension. The symptoms were however associated with a significant weight loss despite good appetite and adequate feeding. There were no associated respiratory and urinary symptoms. The past medical history was remarkable in the sense that she had initially presented to a general hospital where she was investigated and treated for dysentery with a course of metronidazole, co-trimoxazole and hyoscine bromide for 6 weeks without any appreciable improvement. There was no history or signs of past abdominal surgery. Patient is the first of three children to both monogamous parents. The parents are Nigerians and there was no history of similar illness in any member of the family.

On examination, she was afebrile, anicteric, mildly pale, weighed 19kg, not irritable or in respiratory distress, not dehydrated or had peripheral oedema. There was no peripheral lymphadenopathy, skin desquamation or skin discolorations. The mucous membranes and nails were normal. Mild tenderness was elicited in the peri-umbillical region but no palpable abdominal mass, hepatomegaly or splenomegaly. Rectal examination was painful, no palpable rectal mass. The rectum appeared to be narrowed and the examination finger was stained with frank blood.

**Figure 1:**
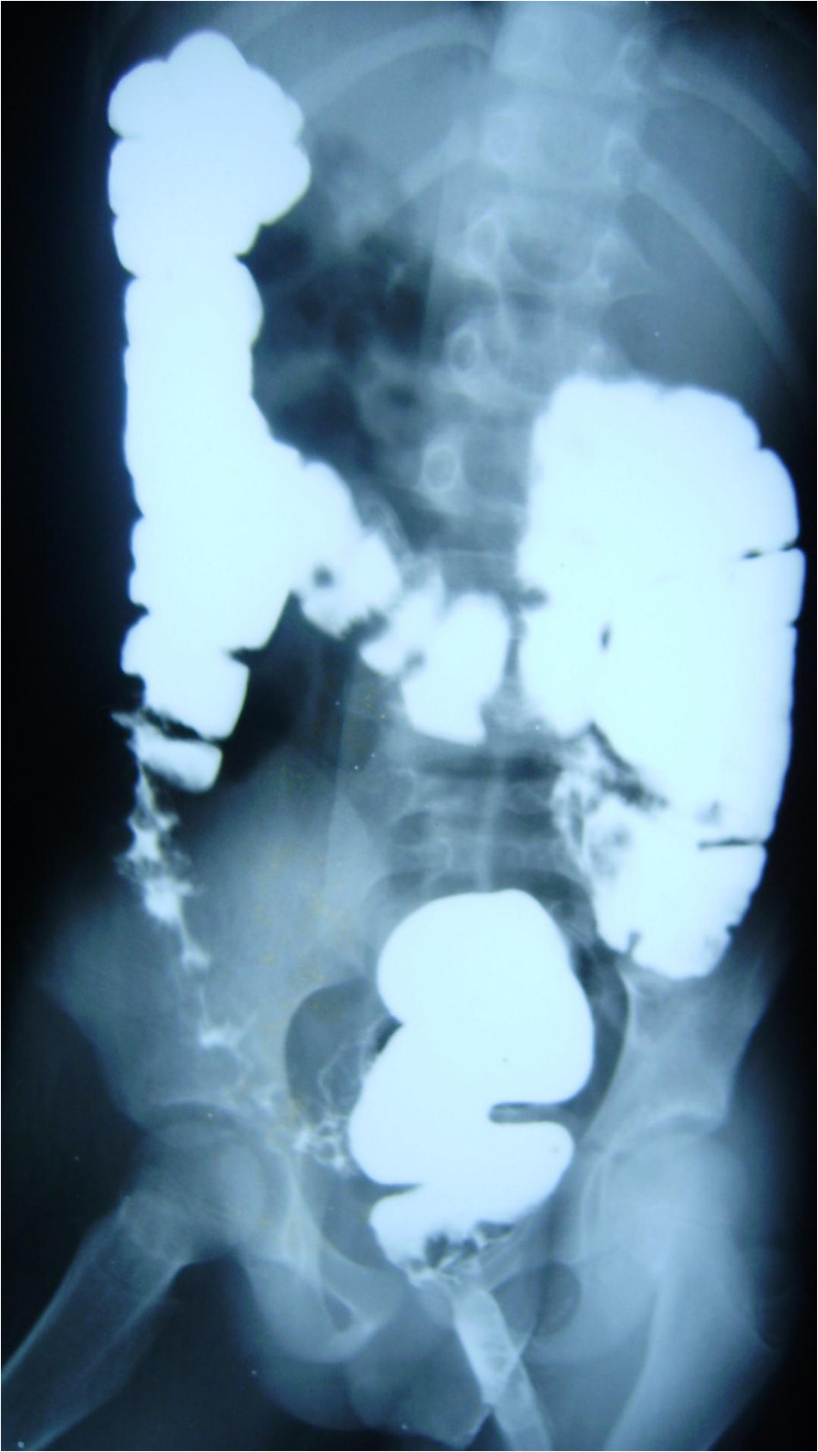
Barium enema showed dilatation of the sigmoid and descending colon, persistent narrowing of the rectum with effacement of the mucosal pattern that was replaced by thumb printing appearances.

**Figure 2:**
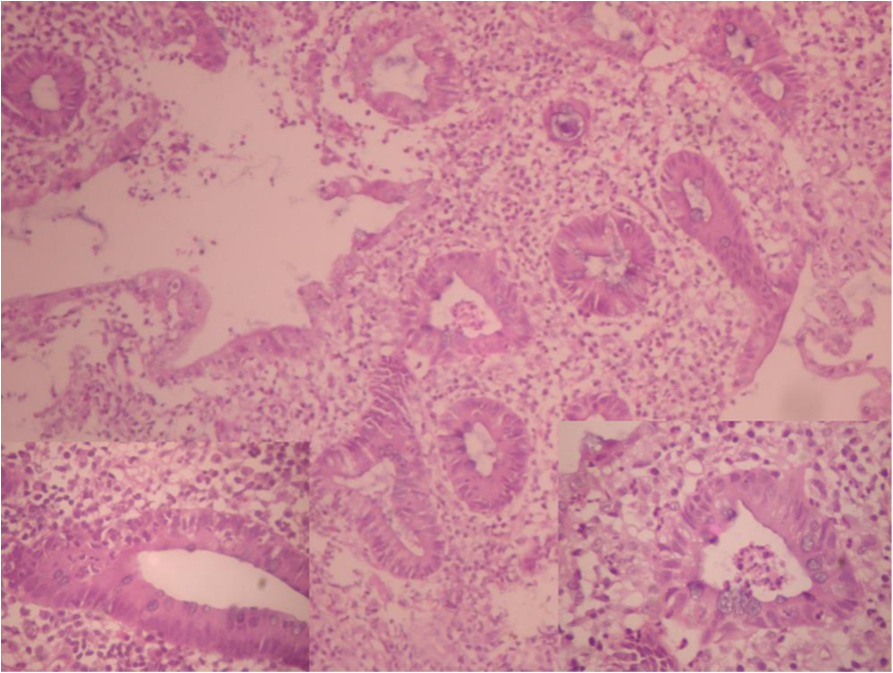
**Photomicrograph of UC (rectal mucosa) showing intense inflammation with disordered crypts and evidence of cryptitis and crypt abscess.** H & E (magnification x 200).

The patient was admitted and investigated for causes of lower gastrointestinal bleeding. The investigations revealed Hb of 10g/dL, white blood cell count of 19,400/mm^3^ with neutrophil differential of 61%, lymphocyte-32% and monocyte-7%. The ESR was elevated to 34mm/hr and serum protein significantly reduced with hypoalbuminaemia of 21g/dL. The liver function test and electrolyte with urea were essentially normal. The stool and urine cultures yielded no growth after 48 hours of incubation. No eggs, ova or intestinal parasites were seen on stool microscopy. Patient was commenced on a high protein diet and all antibiotics discontinued for 10 days. While the symptoms persisted, barium enema was requested which showed dilatation of the sigmoid and descending colon in association with persistent narrowing of the rectum and effacement of the mucosal pattern that was replaced by thumb printing appearances (Figure 1). These findings were suggestive of UC. Colonoscopy and rectal biopsy were performed later. The colonoscopy showed inflammatory changes extending from the anal opening up to the visible part of the descending colon. The bowel mucosa was erythemous and oedematous, with effaced vascular pattern. Tissue biopsy was granular and friable. The histology of the rectal tissue biopsy confirmed UC as shown in Figure 2.

Patient was commenced on sulfasalazine 50mg/kg/day in two divided doses and gradually increased to 60mg/kg/day after a week treatment as it was well tolerated. Patient was in remission until 6 months follow up. However, she defaulted from the clinic for about 6 months but continued taking her medications at home for another 5 months. She presented again with bleeding diarrhoea after stopping her medications for about a month. Sulfasalazine was re-commenced at the same dose plus prednisolone 1 mg/kg/day in two divided doses. Patient responded very well to the new regime and now in remission. She is being followed at the outpatient clinic every 4 weeks.

## Discussion

UC is known to affect children and adults globally. However, it is less common in Africa probably due to under-diagnosis, misdiagnosis or low racial distribution. Few cases of UC in adults have been reported in South Africa [15], Uganda [16] and Sudan [17]. Lack of reported cases in African children therefore underscores the importance of this current case report.

A major challenge in the management of UC in developing countries is making an accurate diagnosis. Our patient was presumptively treated for amoebic dysentery at a general hospital. In spite of persistent symptoms after 6 weeks of antibiotic therapy, UC was not suspected by her physician. Thus, a high index of suspicion may be required for early diagnosis of UC which should be considered as a differential diagnosis of blood stained chronic diarrhoeal diseases in children.

Presently, there is no permanent medical cure for UC [1,2]. The general goals of treatment in children are to control symptoms of the disease with minimal adverse effects of the medicines used and to achieve normal functioning of the patient [1,2]. A multidisciplinary approach has been suggested for effective management of UC in children [2]. Patient should be treated and followed up jointly by a team consisting of a paediatric gastroenterologist, paediatric surgeon, child psychiatrist, clinical psychologist and social worker. The intensity of treatment is dependent on the severity of the disease [1]. Less than 5% of children with UC may present predominantly with extraintestinal manifestations, such as growth failure; arthropathy; dermatological, genitourinary or pulmonary manifestations; coagulopathy; or liver disease [1,2]. However, none of these symptoms was manifested in the patient. Based on the symptoms and signs of bloody diarrhoea, abdominal cramps, urgency to defecate, abdominal tenderness, weight loss and mild anemia at presentation, and colonoscopy with histologic findings, the patient was diagnosed of moderate UC [2]. Mild to moderate UC is usually treated on an outpatient basis. Admission becomes inevitable upon failure of maximal outpatient therapy or progression to severe disease [1,2].

The patient was managed on outpatient basis after initial investigation and stabilization on admission. She was commenced on paediatric medical regimen for UC consisting of low residue diet, antimotility and sulfasalazine (a first line medicine) [1,2]. Sulfasalazine is known to treat UC effectively and prevents recurrence [1,2]. Its prolonged use, even during remission, has been recommended in children [1]. However, hypersensitivity adverse reaction to the sulfa component of sulfasalazine is a major limitation to its use as a first line medicine which may occur in 10-20% of patients [2]. Fortunately, the medicine was well tolerated by the patient. On rare occasions, sulfasalazine can exacerbate the symptoms and signs of UC which may prompt patient to self discontinue the medication. On the contrary, there was a tremendous improvement in the patient following the use of sulfasalazine. The medication was self discontinued as both the patient and the parents felt a permanent cure had been achieved after 11 months of remission. Lack of adequate counseling and psychosocial support might have contributed to the poor drug compliance exhibited by the patient at a later stage of treatment. The role of a clinical psychologist is to promote the psychological wellbeing of the patient and enable her to adjust to her daily normal life activities. The multidisciplinary approach to managing this type of patients is important and equally necessary when managing other chronic childhood illness. Unfortunately, the number of clinical psychologists and social workers in Nigeria is just a handful and are confined to academic institutions and tertiary health care facility.

Prednisolone is a second line medicine that was included in the treatment when the patient re-presented to our hospital. The use was justified by the slow response to sulfasalazine. However, both medicines were able to abate the symptoms and signs of the recurrent UC without any adverse effects. Corticosteroids are known to control acute flares of UC effectively but less effective at maintaining long term remission [2,18]. The numerous adverse effects of prolong use of corticosteroids also preclude their maintenance use during UC remission. We, therefore plan to taper off the dose of prednisolone over time and maintain the patient on sulfasalazine after achieving a prolonged remission.

Clinic default and poor medication compliance is a common problem of children with chronic diseases [19]. Poor follow-up has been reported in South African adults with UC [14]. Further default and poor medication compliance may put the patient at risks of progressing to fulminant colitis or becoming refractory to medical therapy [1,2]. Approximately 5-10% of such patients may require acute surgical intervention [2,20]. Surgery should, however, be seen as a complementary to medical therapy and as a means of preventing complications [20].

## Conclusion

This is the first time UC is reported in an African child. Under-diagnosis and misdiagnosis may have accounted for lack of reports on this subject from Africa. Ingenuity may therefore be required for early diagnosis. UC should be suspected in childhood bloody chronic diarrhoeal diseases and patient should be investigated as such. Optimal management is required to achieve long term remission on medical therapy with minimal adverse effects.

## Consent

A written informed consent was obtained from the patient’s legal guardian for publication of this case report and any accompanying images.

## Competing interest

Nil.

## Authors' contributions

IOS was the Pediatric gastroenterologist that managed the patient, conceived and designed the report. KAO participated in the management of the patient and the design of the report. CAO carried out the colonoscopy and biopsy assisted by IOS. FBA carried out the histopathologic evaluation of specimens and interpreted the patient samples. OFN guided and provided essential comments during production of the manuscipt. All the authors read and approved the final manuscript.
